# Youssef's Syndrome following Cesarean Section

**DOI:** 10.1155/2015/605325

**Published:** 2015-09-17

**Authors:** Ozer Birge, Ertugrul Gazi Ozbey, Mustafa Melih Erkan, Deniz Arslan, Ilkan Kayar

**Affiliations:** ^1^Department of Gynecology and Obstetrics, Nyala Sudan Turkey Training and Research Hospital, Nyala, Darfur, Sudan; ^2^Department of Urology, Nyala Sudan Turkey Training and Research Hospital, Nyala, Darfur, Sudan; ^3^Department of Gynecology and Obstetrics, Celal Bayar University Hospital, Manisa, Turkey; ^4^Department of Gynecology and Obstetrics, Osmaniye State Hospital, Osmaniye, Turkey

## Abstract

Youssef's syndrome is characterized by cyclic hematuria (menouria), absence of vaginal bleeding (amenorrhea), and urinary incontinence due to vesicouterine fistula (VUF), the least common of the urogynecological fistulas. Youssef's syndrome has a variable clinical presentation. A vesicouterine fistula is an abnormal pathway between the bladder and the uterus. The most common cause is lower segment Cesarean section. Conservative treatment may be appropriate in some cases, but surgery is the definitive treatment. Vesicouterine fistula should be suspected in cases presenting with urinary incontinence even years after Cesarean section. Diagnostic tests as well as necessary appropriate surgery should be performed on cases with suspected vesicouterine fistula. We present a 40-year-old multiparous woman with vesicouterine fistula after primary Cesarean section; she presented with urinary incontinence, hematuria, and amenorrhea 1 year after the birth. Here, we discuss our case with the help of previously published studies found in the literature.

## 1. Introduction

A vesicouterine fistula is an abnormal pathway between the bladder and the uterus. The first case was reported by Knipe and colleagues in 1908. Vesicouterine fistula is the least common of all the urogenital fistulas, representing 1–4% of all cases [1]. The vast majority of vesicouterine fistulae are secondary to iatrogenic causes, the most common being lower segment Cesarean section [2]. The less frequent causes include induced abortion, dilatation and curettage, vaginal birth after previous Cesarean section, obstructed labor, forceps delivery, placenta percreta, migrated intrauterine contraceptive device, and brachytherapy [2]. The main symptoms of VUF (vesicouterine fistula) are urinary incontinence, cyclic hematuria (menouria), amenorrhea, and urinary tract infection. Most of the cases present in a delayed fashion, from weeks to years after the inciting event [3]. In such cases, the diagnosis is mainly established by clinical detection of urine or dye passing through the external cervical os or by means of a hysterosalpingogram or micturating cystourethrogram, which will demonstrate the fistulous communication [3]. Conservative treatment may be appropriate in some cases, but surgery is the definitive treatment. Transabdominal, laparoscopic, or robotic methods can be used. We present a case with vesicouterine fistula after primary Cesarean section; the patient presented with urinary incontinence, hematuria, and amenorrhea 1 year after giving birth.

## 2. Case Presentation

**Figure 1:**
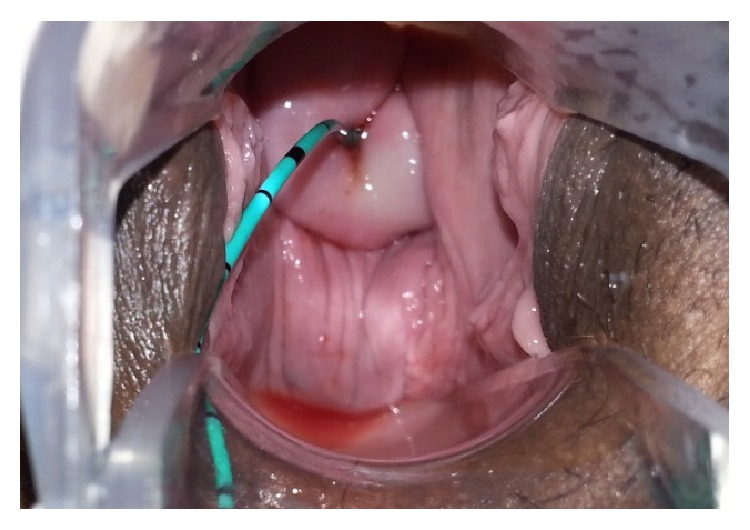
Fluid outflow consistent with hematuria from external os on cervical speculum examination.

**Figure 2:**
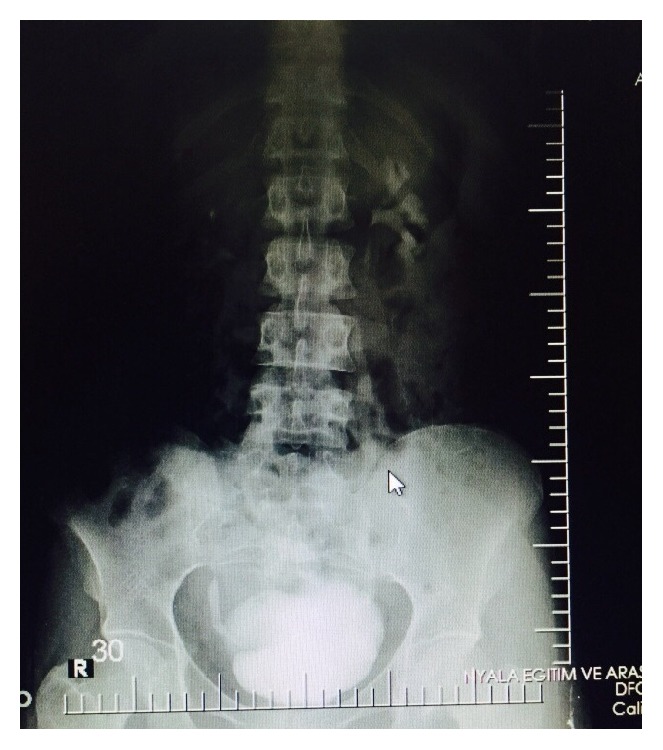
Intravenous pyelography image of urinary system.

**Figure 3:**
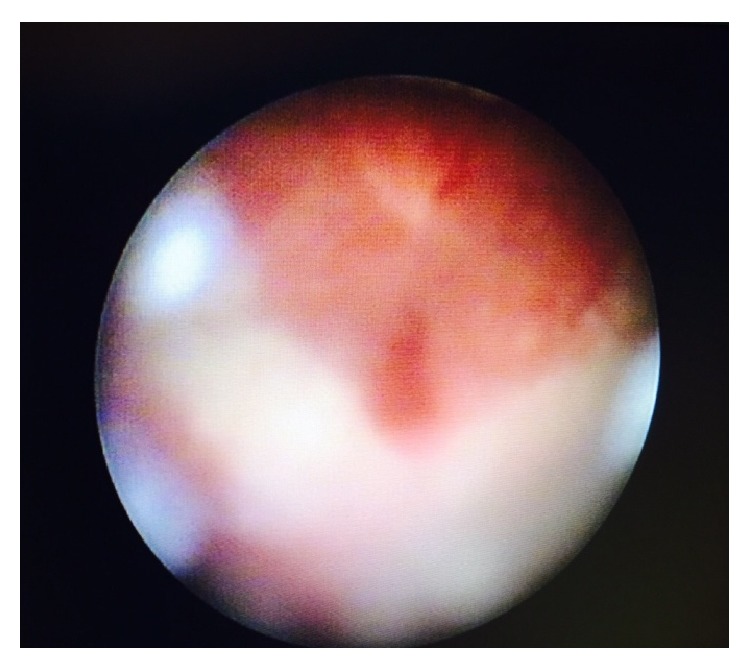
Cystoscopy view of fistula focus on posterior bladder wall.

**Figure 4:**
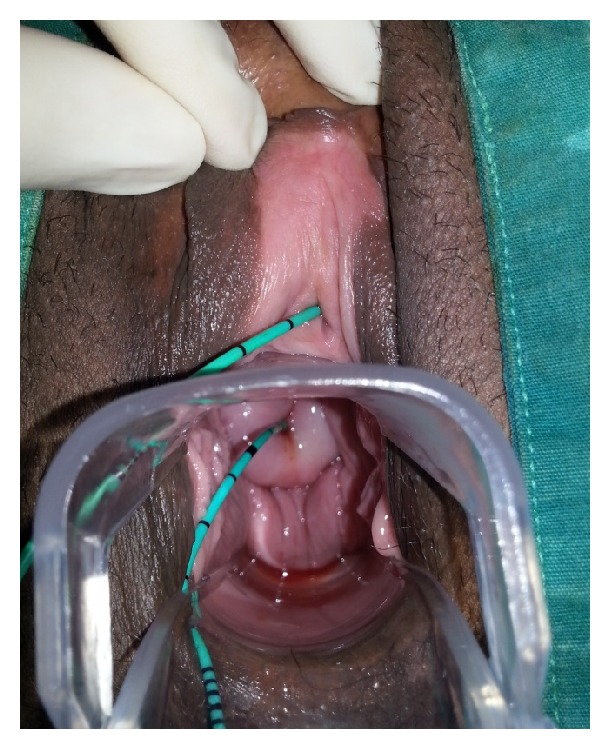
Exit of catheter stent through cystoscope to the fistula focus in bladder from cervical external os.

A 40-year-old multiparous female patient presented to our clinic with urinary incontinence, hematuria, and amenorrhea a year after giving birth by Cesarean section due to fetal distress in our clinic. Patient history had no highlights except a lower segment Cesarean section by Pfannenstiel incision due to fetal distress in previous year. Transvaginal USG (ultrasonography) showed normal uterine and ovary structures. Noticeable urine smell showed that the case had moist vulvar area and speculum examination showed a leakage consistent with hematuria that flowed from cervical os (Figure 1). After administering methylene blue through insertion of a urethral Foley catheter, there was an active outflow of methylene blue from cervical os. IV (intravenous) pyelography showed no anatomical defects or significant leakage (Figure 2). Cystoscopy found the fistula focus on posterior bladder wall (Figure 3). During cystoscopy, catheter pushed through the fistula on posterior wall of bladder came out of cervical external os (Figure 4). After informing the patient and her relatives about the clinical situation thoroughly, laparotomy was decided. Since the patient wanted to keep her fertility intact, an extraperitoneal approach by Pfannenstiel incision was used to reach the bladder. The bladder was removed using O'Connor method and following the detection of fistula focus between uterus and bladder, necrotic fistula tract was removed and bladder and uterine mucosa were sutured shut by 2 layers of 2-0 polyglycolic sutures. Leakage control was done by administering sterile saline solution to the bladder and no leakage was seen in suture lines. An 18-Fr Foley catheter was placed into urethra and a drain was placed on extraperitoneal area. The drain placed on abdomen was removed 5 days after and urethral Foley catheter was removed 14 days after the spontaneous urination was normal and no leaks were detected.

## 3. Discussion

In 1957, Youssef described a syndrome comprising of cyclic hematuria, amenorrhea, menouria, and complete urinary incontinence in a patient who had lower segment Cesarean section (LSCS) [4]. The VUFs are among the least common urogynecological fistulas. The VUF also occurs following high vaginal forceps-aided delivery, external cephalic version, curettage or manual removal of the placenta, placenta percreta, myomectomy, uterine rupture due to obstructed labor, uterine artery embolization, perforation of an intrauterine device, and brachytherapy for carcinoma of cervix. The LSCS is the single most common cause of VUF [3]. Amenorrhea, cyclic hematuria without urinary incontinence in combination with a history of LSCS, has been described as pathognomonic of VUF [5]. The clinical presentation is often nonspecific and findings on examination classically used to depict the fistula may be negative, leading to considerable delay in diagnosis [6]. The VUF may not manifest with constant urinary incontinence because of a functional sphincter at the internal uterine os. Urinary incontinence occurs if the level of the VUF is at or below the internal os or if the os is incompetent [5]. In our case scanty urine leak occurred even in the presence of competent os with fistula communicating with uterus above isthmus.

The diagnosis of VUF is often confirmed by imaging studies and cystoscopy. Cystoscopy, even when repeated, can fail to confirm the fistula [4]. Methylene blue instilled into the uterine cavity or through the urethra or through catheterization of a visible lesion in the bladder wall can confirm the fistula. This test, however, does not show directly the fistulous tract and its specific location. Moreover, this test can be negative in patients with a long and tortuous tract [4]. In radiological studies, both cystography and hysterography have been used in the diagnosis of VUFs. In Tancer's review of published reports, he found that hysterography was the most reliable diagnostic technique [5]. Intravenous urography can show the fistula when contrast medium enters the vagina, but distinguishing vesicovaginal and vesicouterine fistulae is difficult.

Although VUFs are difficult to diagnose using USG, Park et al. reported that sonography can demonstrate the fistulous tract as double echogenic lines between the endometrium of the anterior wall of the uterine body and the mucosal layer of the posterior wall of the bladder [3]. However, sonography has inherent difficulty in differentiating the VUF tract from different patterns of a noncomplicated Cesarean scar [6]. Also transvaginal sonography is recently being used for diagnosis [7]. Magnetic resonance imaging has now become the first choice in the investigation of fistulas [8, 9].

Treatment methods include expectant management with long-term bladder catheterization, medical treatment, and surgery. Spontaneous closure of vesicouterine fistula has been reported [10]. Medical treatment involves induction of amenorrhea to aid in fistula healing [11]. Oral contraceptives, progestational agents, and gonadotropin releasing hormone analogs have been used to induce amenorrhea [12]. Surgery is the definitive method of treatment. It can be performed transabdominally, endoscopically, and robotically. The transvaginal approach is not preferred because of the higher location and complexity of the fistulas.

Transabdominal repair can be performed by extraperitoneal or retrovesical (O'Connor) technique [13]. Disadvantages of the transabdominal route are increased morbidity, long hospital stay, and increased blood loss; these can be overcome by using the endoscopic and robotic approaches [7]. For the laparoscopic approach, the surgeon should be skilled in fistulous tract dissection and intracorporeal suturing. Robotic-assisted surgery overcomes some of the difficulties related to the laparoscopic approach by better imaging and ease of intracorporeal suturing [14].

In our case, fistula focus between the bladder and lower segment of uterus was found using O'Connor technique through extraperitoneal abdominal incision and mucosa of uterus and bladder were repaired using long-term absorbable suture materials. Extraperitoneal drainage catheter placed in abdomen was removed after 5 days and urethral Foley catheter was removed after 14 days following operation. No urine leakage was detected and spontaneous urination was normal.

## 4. Conclusion

Vesicouterine fistulas are uncommon, yet they are becoming more prevalent due to changes in modern obstetrical care. They should always be kept in mind in patients with a history of Cesarean section or who have experienced a gynecological procedure associated with signs of hematuria and/or urinary leakage.
